# Functional consequences of diminished myocardial oxygen delivery per beat in experimental heart failure

**DOI:** 10.1007/s00395-026-01186-5

**Published:** 2026-05-19

**Authors:** Salman I. Essajee, Matthew J. Eden, Victoria E. Sturgess, Gregory M. Dick, Selina M. Tucker, Cooper M. Warne, C. Alberto Figueroa, Daniel A. Beard, Johnathan D. Tune

**Affiliations:** 1 Department of Physiology and Anatomy, University of North Texas Health Science Center, 3500 Camp Bowie Blvd., Fort Worth, TX 76107 USA; 2 Section of Vascular Surgery, Department of Surgery, University of Michigan, Ann Arbor, MI USA; 3 Department of Biomedical Engineering, University of Michigan, Ann Arbor, MI USA; 4 Department of Molecular and Integrative Physiology, University of Michigan, Ann Arbor, MI USA

**Keywords:** Coronary blood flow, Perfusion-contraction matching, Coronary autoregulation, Pacing-induced heart failure, Swine

## Abstract

This investigation was designed to test the hypothesis that heart failure (HF) attenuates coronary vasodilation and autoregulation and that deficits in contractile function are proportionally related to reductions in the volume of myocardial perfusion and oxygen delivered per beat. Utilizing a pacing-induced model of HF in Ossabaw swine, we determined that chronic pacing at 180 beats/min for ~ 4 weeks significantly reduced baseline coronary flow by ~ 45% (*P* = 0.01), lowered myocardial oxygen consumption (MVO₂) (*P* = 0.07) and systolic wall thickening by ~ 30% (*P* < 0.01), and increased left ventricular end-diastolic pressure ~ 160% (*P* < 0.05). Coronary flow responses to Regadenoson (0.4 mg) and cardiac pacing (180 beats/min) were significantly attenuated in swine with pacing HF (*P* ≤ 0.01). Control swine displayed relatively strong autoregulatory capability with coronary flow decreasing ~ 10% (*P* = 0.26) as blood pressure was pharmacologically reduced from 120 to 60 mmHg. Alternatively, coronary flow decreased ~ 40% (*P* = 0.01) over the same pressure range in pacing HF swine. Regional wall thickening and stroke volume declined once myocardial oxygen delivery fell below ~ 1.0 µL O₂/g/beat. These findings indicate that adaptations in the coronary microcirculation in pacing HF attenuate coronary metabolic and autoregulatory capacity and that subsequent functional deficits are related to reductions in the volume of myocardial perfusion and oxygen delivered per beat.

## Introduction

Heart failure is a clinical syndrome characterized by cardiac dysfunction that results in insufficient oxygen delivery to peripheral tissues [1, 2]. The multifactorial etiology of heart failure is a complex interplay between genetic, environmental, and comorbid factors that promote the development of either heart failure with reduced ejection fraction (HFrEF) or heart failure with preserved ejection fraction (HFpEF) [1, 3]. There is a growing body of evidence to support that heart failure is strongly associated with impaired coronary vasodilator capacity, i.e. coronary flow reserve [4–8]. This underlying coronary microvascular dysfunction is a powerful predictor of major adverse cardiovascular events (MACE) [1, 7–9] and has been directly associated with key features of the failing heart including myocardial fibrosis, capillary rarefaction, and myocyte death [10–12]. While prior studies implicate impaired coronary microvascular reactivity in the initiation and/or progression of the heart failure phenotype, understanding of the regulation of coronary blood flow in the setting of heart failure remains quite poor [7, 8, 13].

Under normal physiological conditions, myocardial perfusion is dictated by changes in myocardial oxygen consumption (MVO_2_) and underlying levels of cardiac contractile function [14–21]. Prior studies in human subjects with heart failure suggest baseline coronary blood flow is normal to modestly diminished in HFrEF [22, 23] whereas flow is normal to slightly elevated in HFpEF [24, 25]. Regardless, reductions in coronary flow reserve in both phenotypes are proposed to constitute a vicious cycle between impaired mechanisms of coronary microvascular control and the advancement of overt myocardial failure [13]. Recent evidence from our laboratory indicates that the degree of coronary microvascular dysfunction is directly proportional to deficits in contractile function and MVO_2_ relative to reductions in the volume of myocardial perfusion per beat [16]. However, the extent to which the impairment of myocardial perfusion and oxygen delivery determines functional and metabolic consequences of heart failure has not been defined.

The purpose of this study was to investigate interrelationships between coronary blood flow, MVO_2_, and regional contractile function in the setting of heart failure. Experiments were designed to test the hypothesis that heart failure attenuates coronary vasodilation and autoregulatory capacity and that subsequent deficits in function are directly related to reductions in the volume of myocardial perfusion and oxygen delivered per beat [16, 21, 26]. Utilizing our model of pacing-induced heart failure in Ossabaw swine [12] we examined coronary and cardiac responses to the adenosine analog Regadenoson, to increases in heart rate, and to a wide range of systemic arterial pressures. Findings provide novel insight into the consequences of coronary microvascular dysfunction that could contribute to the progression of the heart failure phenotype.

## Methods

Experiments performed in this investigation were approved by the University of North Texas Health Science Center Institutional Animal Care and Use Committee and performed in accordance with the Guide for the Care and Use of Laboratory Animals (NIH Publication No. 85–23, Revised 2011) [27]. Ossabaw swine (~ one-year of age) were fed a standard chow for the entire duration of the study (~ 2200 kcal/day; 5L80 Purina TestDiet (n = 10)). Swine were randomized to either a control (n = 5; (n = 3 female)) or pacing-induced heart failure (pacing HF; n = 5; (n = 2 female)) group and subjected to the procedures outlined below. Upon completion of the experimental protocols, anesthetized animals were humanely euthanized by electrical fibrillation and excision of the heart. Coronary arteries isolated from both control (n = 5) and pacing HF (n = 5) swine reported in this study were utilized for assessment of transmural coronary biomechanics in another recently published study from our laboratory [28].

### Chronic Instrumentation and pacing protocol

Swine designated for the pacing HF group (n = 5) were sedated with Telazol (tiletamine-zolazepam, 5 mg/kg, sc), xylazine (2.2 mg/kg, sc), and ketamine (3.0 mg/kg, sc) and following endotracheal intubation, anesthesia was maintained with isoflurane (3–5%). As previously described by our laboratory [12], utilizing sterile technique, a lateral thoracotomy was performed and a pacemaker lead secured to the surface of the right ventricular free wall. The lead was tunneled subcutaneously, connected to a Biotronik pulse generator, and the chest closed in layers. After recovery from surgery (~ 5–7 days), the pulse generator was activated and programmed to increase heart rate to 180 beats/min, confirmed by electrocardiogram, for ~ 4 weeks.

### Acute surgical preparation

Following the completion of the ~ 4 week right ventricular pacing protocol, pacemakers were turned off and control (n = 5) and pacing HF (n = 5) swine were anesthetized with Telazol (tiletamine-zolazepam, 5 mg/kg, sc), xylazine (2.2 mg/kg, sc), and ketamine (3.0 mg/kg, sc). Anesthesia was maintained with buprenorphine (0.03 mg/kg, sc) and α-chloralose (100 mg/kg, iv) and mechanical ventilation with supplemental oxygen was utilized to maintain oxyhemoglobin saturation > 95% and an end tidal CO_2_ of ~ 40 mmHg; measured by aural pulse oximetry and inline capnography respectively. Bilateral femoral cut downs were performed, and catheters placed in both femoral arteries and veins. One femoral artery catheter provided continuous blood pressure and heart rate monitoring and both veins were used for intravenous administration of various agents. The other femoral artery catheter was utilized for arterial blood gas sampling. A left lateral thoracotomy was then performed, and the left anterior descending coronary artery (LAD) was isolated, and a perivascular flow transducer (Transonic Systems Inc. Ithaca, NY, USA) placed around the artery. A catheter was introduced into the lumen of the left ventricle (LV) and secured with a purse string suture. A catheter was then introduced into the interventricular vein to obtain coronary venous samples from the LAD perfusion territory. Hemodynamic parameters, coronary blood flow and LV pressure were continuously acquired using IOX acquisition software (EMKA Technologies, Falls Church, VA, USA).

### Experimental protocol

Following surgical preparation and an ~ 15–30 min stabilization period, baseline hemodynamic, blood gas and echocardiographic data were obtained. Subsequently, coronary blood flow responses to the intravenous administration of the adenosine A_2a_ receptor agonist Regadenoson (0.4 mg) were recorded. After hemodynamic variables returned to baseline levels (~ 15 min), blood pressure was subsequently increased by titrating a systemic infusion of phenylephrine (10 µM solution) to achieve a sustained average blood pressure of ~ 140 mmHg. Once hemodynamics and coronary flow were stable at this pressure, arterial and coronary venous blood samples were collected and echocardiographic imaging of LV obtained. Mean blood pressure was subsequently reduced in ~ 20 mmHg stepped decrements from ~ 140 mmHg to ~ 40 mmHg by lowering doses of phenylephrine and by increasing infusion of sodium nitroprusside (1 µM solution) to achieve each desired pressure. Blood samples and LV imaging were obtained once hemodynamic variables were constant at each pressure (~ 5–10 min at each pressure). After data were recorded at the lowest pressure, blood pressure was returned to baseline levels of ~ 100 mmHg. After hemodynamic variables returned to baseline levels (~ 15 min), coronary and cardiac responses to pacing-induced increases in heart rate (180 beats/min) were recorded.

As previously reported by our laboratory and others [29, 30], closed-loop autoregulatory gain (Gc) was calculated from the following formula: $Gc = 1 - ((\Delta F/F)/(\Delta CPP/CPP))$, where ΔF/F = ((F_120_–F_60_)/F_120_) and ΔCPP/CPP = ((CPP_120_–CPP_60_)/CPP_120_) represent the changes from a given flow (F) and coronary perfusion pressure (CPP) over a CPP range of 120 mmHg to 60 mmHg [29]. A Gc value of 1 reflects perfect autoregulation, and values < 0 indicate no autoregulation.

### Echocardiographic imaging

Acquisition of echocardiographic images was performed using a Philips Epiq-7C model machine along with an X-51 cardiovascular probe to acquire 2D images of the LV anterior wall [16]. Images were obtained in open-chest swine via placement of the probe (placed in a gel-filled sheath) directly on the surface of the heart. 2D images to measure anterior LV wall thickness at the end of diastole (DWT: end of QRS complex) and the end of systole (SWT: end of the T-wave) were acquired and averaged from 5 consecutive beats in the apical short-axis view at each stage of the experimental protocol. Ventricular volumes were calculated using the Simpson method. Systolic wall thickening was calculated as the percent change in wall thickness from end diastole to end systole ((SWT-DWT)/DWT) × 100.

### Blood gas and metabolic analyses

Arterial and coronary venous blood samples were collected, sealed and placed on ice for analysis of pH, PCO_2_, PO_2_, hemoglobin saturation, lactate, and oxygen content using an automated blood gas analyzer with CO-oximetry (Prime Plus Vet, Nova Biomedical). Myocardial oxygen consumption (MVO_2_: µL O_2_/min/g) and lactate uptake were calculated using the Fick principle by multiplying coronary blood flow by arterial and coronary venous differences in oxygen content and lactate concentration, respectively. For these calculations, LAD perfusion territory was estimated to be 30% of the total heart weight, as previously described by Feigl [31].

### Statistical analyses

All values are presented as mean ± SEM. Statistical comparisons were performed by t-test or two-way repeated measures analysis of variance (ANOVA; Factor A: Group; Factor B: Condition) with Sigma Plot (version 14.0) as appropriate. When significance (α < 0.05) was found with ANOVA, a Student–Newman–Keuls multiple comparison test was performed to identify differences between conditions. Multiple linear regression analysis was used to compare slopes of response variables (GraphPad Prism 10). If slopes were equivalent, subsequent analysis of covariance (ANCOVA) was used to test for differences in elevation in the relationship between specific response (y-axis) variables relative to changes in the relevant independent (x-axis) variable. Nonlinear regression fits were accomplished by 3rd order polynomial (cubic) fits.

## Results

### Baseline hemodynamic and functional cardiac parameters

Baseline phenotypic and hemodynamic data for control and pacing HF swine are provided in Table 1. Swine with pacing HF tended to have higher body weights (~ 10%; *P* = 0.10), while heart weight and heart to body weight ratios were similar between groups. Although diastolic pressure was ~ 20% higher in pacing HF swine (*P* < 0.05), no differences in baseline mean blood pressure, heart rate, stroke volume, or cardiac outputs were observed between groups. Pacing HF tended to decrease end diastolic volume (~ 15%; *P* = 0.20) but had no effect on ejection fraction (Table 1). In contrast, coronary blood flow was ~ 45% lower in pacing HF vs. control swine which corresponded with ~ 30% reductions in MVO_2_ (*P* = 0.07) and regional systolic wall thickening (*P* < 0.01). LV end diastolic pressure was markedly elevated from 8 ± 1 mmHg in control swine to 21 ± 2 mmHg in pacing HF swine (*P* < 0.05) and lung wet:dry ratios increased by ~ 45% in pacing HF swine (*P* < 0.01).

**Table 1:** Baseline phenotypic characteristics of control and pacing-induced heart failure swine

	Control	Pacing HF
Body weight (kg)	66 ± 3	72 ± 2
Heart weight (g)	221 ± 15	241 ± 15
Heart weight: body weight (g/kg)	3.4 ± 0.3	3.2 ± 0.1
Systolic blood pressure (mmHg)	127 ± 7	130 ± 6
Diastolic blood pressure (mmHg)	87 ± 3	103 ± 6*
Mean blood pressure (mmHg)	102 ± 6	112 ± 6
Heart rate (beats/min)	77 ± 8	81 ± 6
Coronary flow (mL/min/g)	0.63 ± 0.07	0.35 ± 0.07*
MVO_2_ (μL O_2_/min/g)	43 ± 2	30 ± 6
LV wall thickening (%)	28 ± 1	20 ± 1*
LV end diastolic volume (mL)	91 ± 4	78 ± 8
Stroke volume (mL)	49 ± 2	41 ± 5
Cardiac output (L/min)	3.8 ± 0.4	3.4 ± 0.7
Ejection fraction (%)	54 ± 1	52 ± 1
LV end diastolic pressure (mmHg)	8 ± 1	21 ± 2*
Lung wet/dry ratio	5.4 ± 0.1	7.9 ± 0.4*

Values are mean ± SE for Control (n = 5) and Pacing HF (n = 5)

^*^ *P* < 0.05 vs. Control

### Coronary flow responses to pharmacologic and metabolic stimuli

**Fig. 1:**
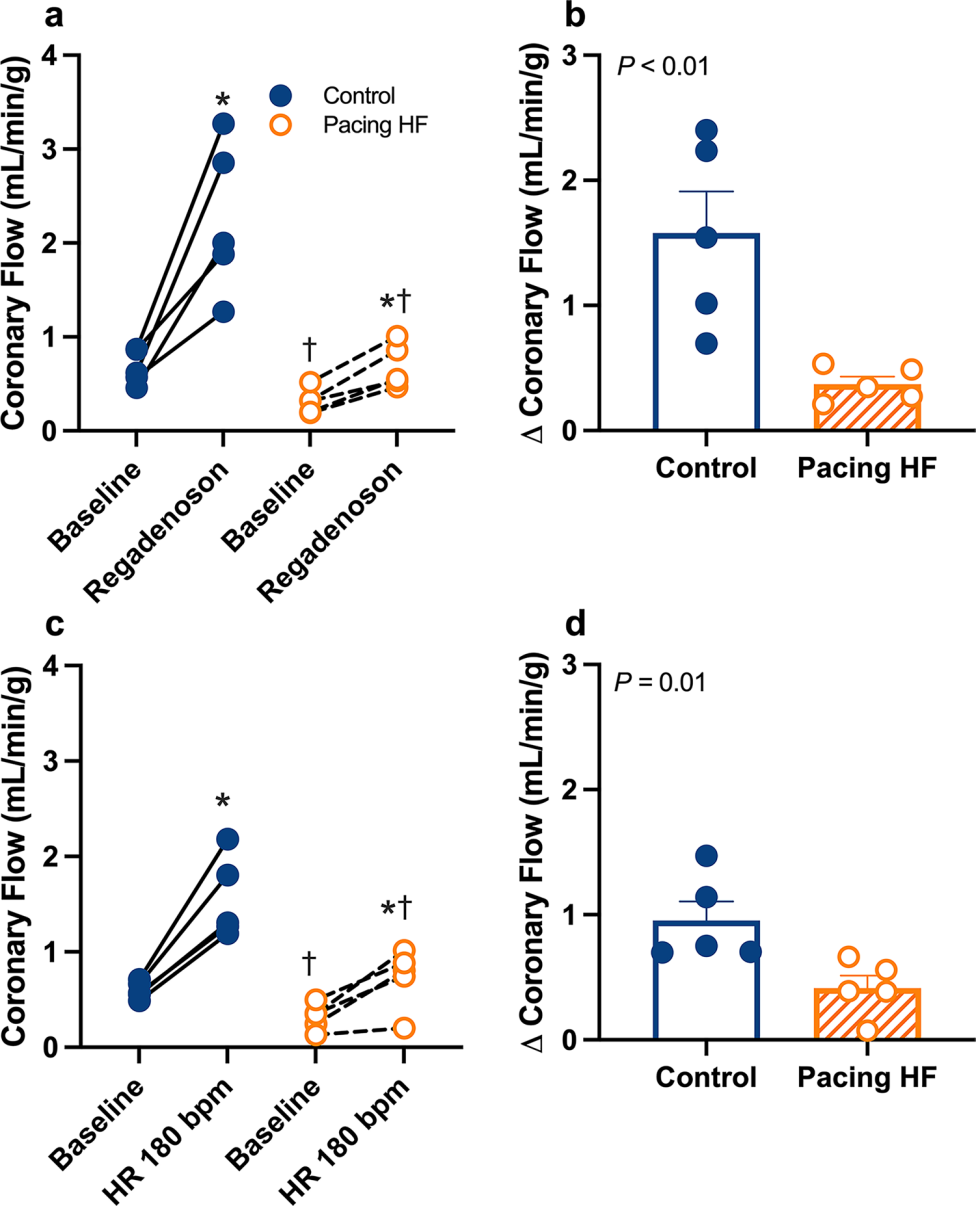
Coronary vasodilator responses to pharmacologic and metabolic stimuli. **a** Coronary blood flow was significantly reduced at baseline and in response to Regadenoson in pacing HF (n = 5) vs. control swine (* *P* < 0.05 vs. Baseline, same group; † *P* < 0.05 vs. Control group, same condition). **b** Absolute (delta) increase in coronary flow to Regadenoson was diminished in pacing HF swine. **c** Coronary blood flow was decreased at baseline and in response to increases in heart rate (180 beats/min) in pacing HF (n = 5) vs. control swine (* *P* < 0.05 vs. Baseline, same group; † *P* < 0.05 vs. control, same condition). **b** Absolute (delta) increase in coronary flow to increases in heart rate was also significantly attenuated in pacing HF swine

The effect of pacing HF on coronary vasodilator capacity is shown in Fig. 1. Systemic administration of Regadenoson (0.4 mg, iv) significantly increased coronary blood flow from 0.67 ± 0.08 mL/min/g to 2.25 ± 0.35 mL/min/g (*P* < 0.01) in control swine and from 0.31 ± 0.06 mL/min/g to 0.68 ± 0.11 mL/min/g (*P* < 0.01) in pacing HF swine. The overall change (delta) in coronary flow to Regadenoson was significantly reduced by pacing HF (*P* < 0.01; Fig. 1b) which corresponds with a decrease in coronary flow reserve from 3.4 ± 0.5 in control swine to 2.3 ± 0.2 (*P* = 0.07) in pacing HF swine. Similarly, coronary responses to cardiac pacing at 180 beats/min increased coronary blood flow from 0.59 ± 0.04 to 1.55 ± 0.19 mL/min/g (*P* < 0.01) in control swine, but only increased flow from 0.32 ± 0.06 to 0.73 ± 0.13 mL/min/g (*P* = 0.01) in the pacing HF group (Fig. 1c); i.e. pacing HF diminished the increase (delta) in coronary flow to pacing from 0.95 ± 0.15 mL/min/g to 0.41 ± 0.10 mL/min/g (*P* = 0.01; Fig. 1d).

### Hemodynamic and cardiac responses to variations in systemic pressure

**Fig. 2:**
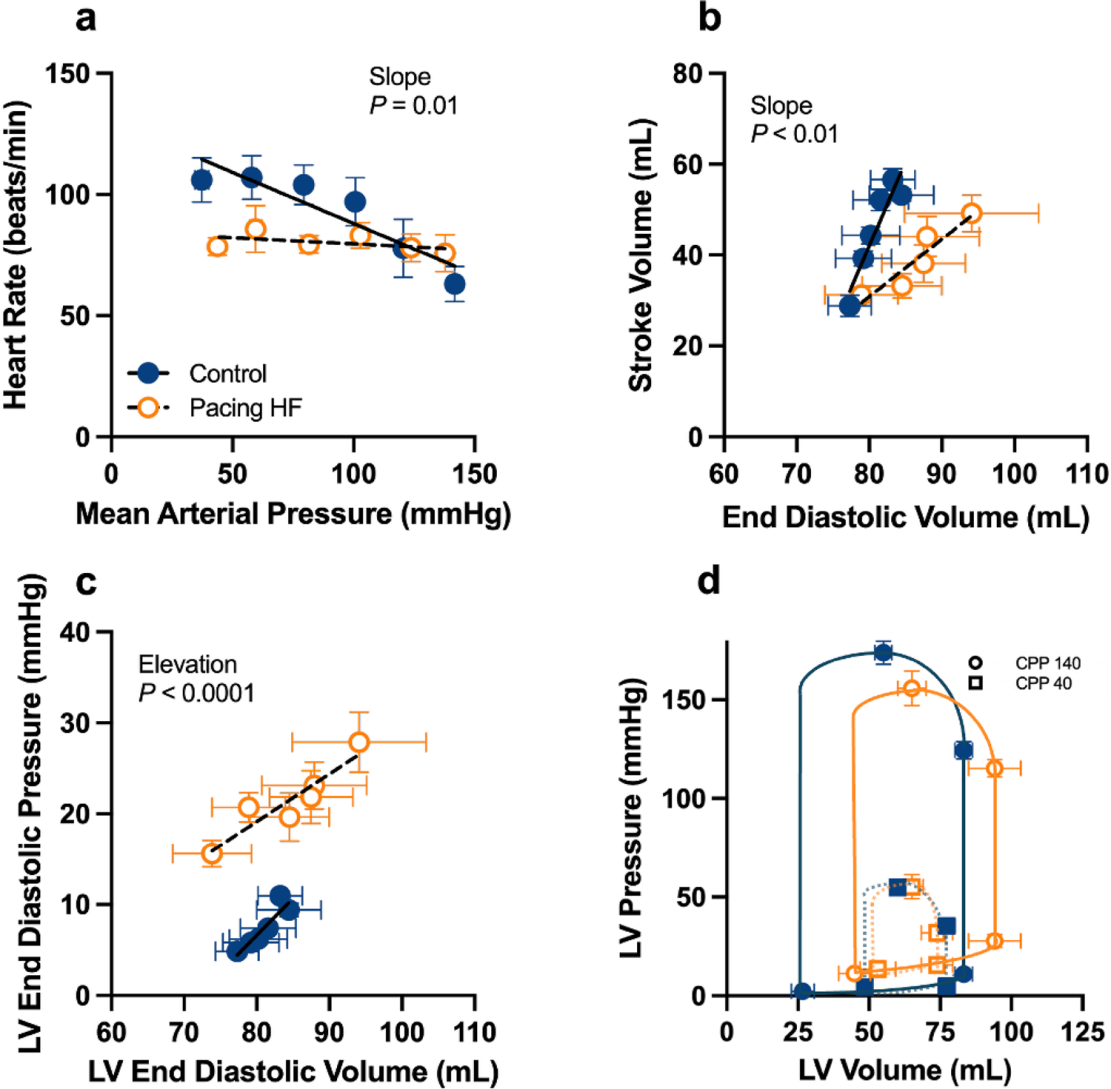
Hemodynamic and functional cardiac responses to reductions in mean arterial pressure from ~ 140 mmHg to ~ 40 mmHg in control (n = 5) vs pacing HF (n = 5) swine. **a** Pacing HF significantly decreased the slope of the relationship between heart rate and mean arterial pressure. **b** Slope of the relationship between LV stroke volume and end diastolic volume was diminished in pacing HF vs. control swine. **c** Pacing HF markedly increased LV end diastolic pressure relative to LV end diastolic volume. **d** Representative LV pressure–volume loops from group averaged data from control and pacing HF swine at blood pressures of ~ 140 mmHg and ~ 40 mmHg

The influence of arterial pressure on hemodynamic, cardiac and metabolic response variables is provided in Table 2. Reductions in mean arterial pressure from ~ 140 mmHg to ~ 40 mmHg elicited a robust ~ 70% increase in heart rate in control swine (Table 2). However, heart rate was unaffected by these changes in pressure in pacing HF swine, such that the slope of the relationship between heart rate and mean blood pressure was significantly attenuated by the induction of heart failure (Fig. 2a, P = 0.01). Assessments of global systolic and diastolic function demonstrated an ~ 65% reduction in the slope of the relationship between stroke volume and LV end diastolic volume (Fig. 2b, P < 0.01) and a marked parallel upward shift in the relationship between LV end diastolic pressure and end diastolic filling volume (Fig. 2c, P < 0.0001). Diminished systolic and diastolic function was associated with a ~ 30 to 50% reduction in MVO_2_ in pacing HF swine, with no difference in myocardial lactate uptake between groups (Table 2). These alterations in cardiac function in pacing HF swine are also evident in examination of average values of LV pressure relative to LV volume (pressure–volume loops) at mean arterial pressures of ~ 140 mmHg and at ~ 40 mmHg (Fig. 2d).

**Table 2:**
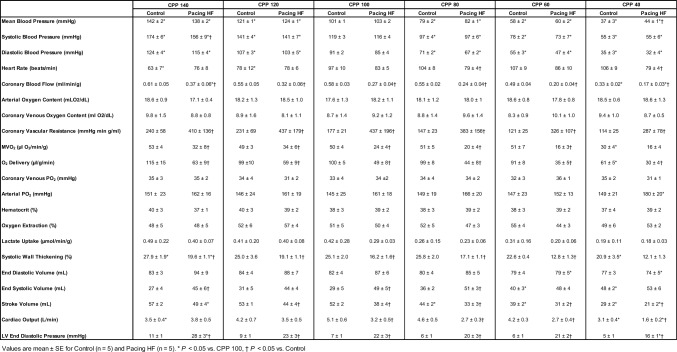
Hemodynamics, cardiac, and metabolic parameters during changes in systemic pressure

Values are mean ± SE for control (n = 5) and Pacing HF (n = 5)

^*^ < 0.05 vs.CPP 100, † P < 0.05 vs. Control

### Coronary flow, contractile function, and metabolism to variations in systemic pressure

**Fig. 3:**
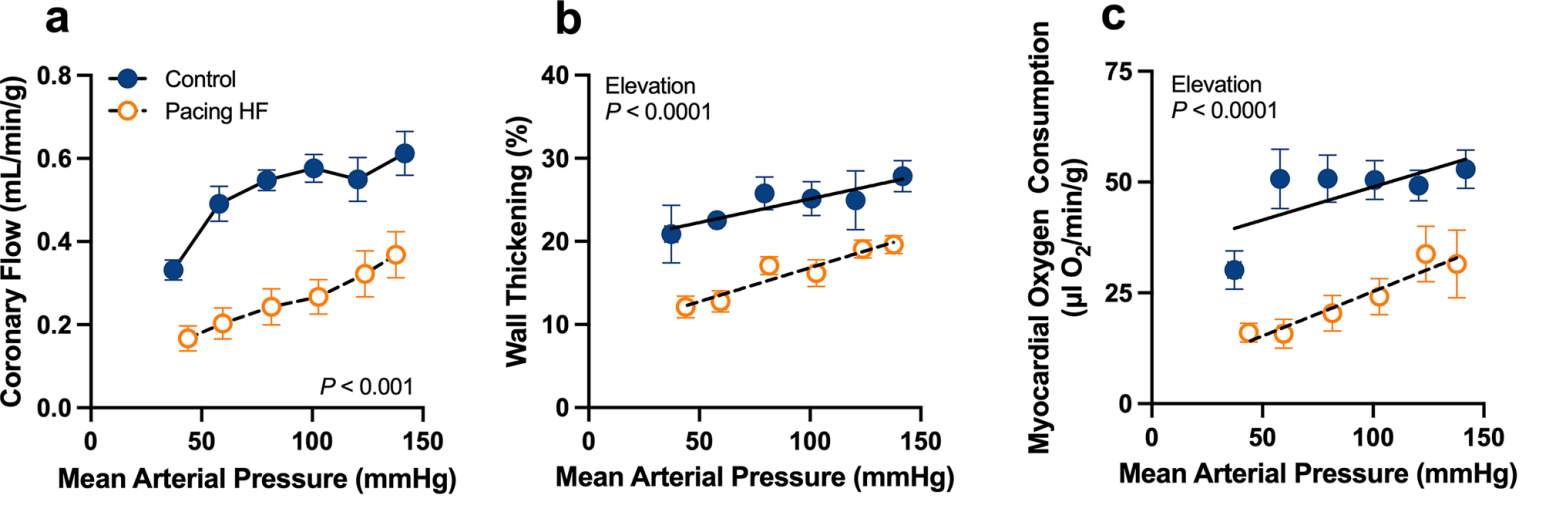
Myocardial perfusion, contraction, and metabolism in response to reductions in mean arterial pressure from ~ 140 mmHg to ~ 40 mmHg in control (n = 5) vs pacing HF (n = 5) swine. **a** Coronary blood flow was significantly diminished across systemic pressures ranging from ~ 140 mmHg to ~ 40 mmHg in pacing HF vs. control swine. **b** Pacing HF resulted in similar reductions in regional systolic wall-thickening and **c** myocardial oxygen consumption as mean arterial pressure was reduced from ~ 140 mmHg to ~ 40 mmHg

The effect of changes in mean arterial pressure (i.e. CPP) on coronary flow, regional contractile function and MVO_2_ are shown in Fig. 3. Coronary flow was significantly depressed (~ 40 – 60%) in the pacing HF group at all pressure steps from 140 to 40 mmHg **(**Fig. 3a**).** Thus, coronary resistance was increased ~ 70% at CPP of 140 mmHg and by ~ 150% at CPP of 40 mmHg in pacing HF swine which resulted in a decrease in myocardial oxygen delivery of ~ 50% across all levels of CPP (Table 2). Control swine displayed relatively strong autoregulatory capability with coronary flow decreasing ~ 10% (*P* = 0.26) as CPP was reduced from 120 to 60 mmHg. Alternatively, coronary flow decreased ~ 40% (*P* = 0.01) over the same pressure range in pacing HF swine (Table 2). These group changes in coronary pressure-flow autoregulation corresponded with autoregulatory gain (Gc) = 0.86 ± 0.24 (control) vs. 0.34 ± 0.18 (pacing HF) (*P* = 0.05; one-tailed t-test). Reductions in coronary flow and autoregulatory capability in the pacing HF group were directly associated with significant parallel downward shifts in the relationships between regional wall-thickening (Fig. 3b; *P* < 0.0001) and MVO_2_ (Fig. 3c; *P* = 0.0035) relative to mean arterial pressure.

**Fig. 4:**
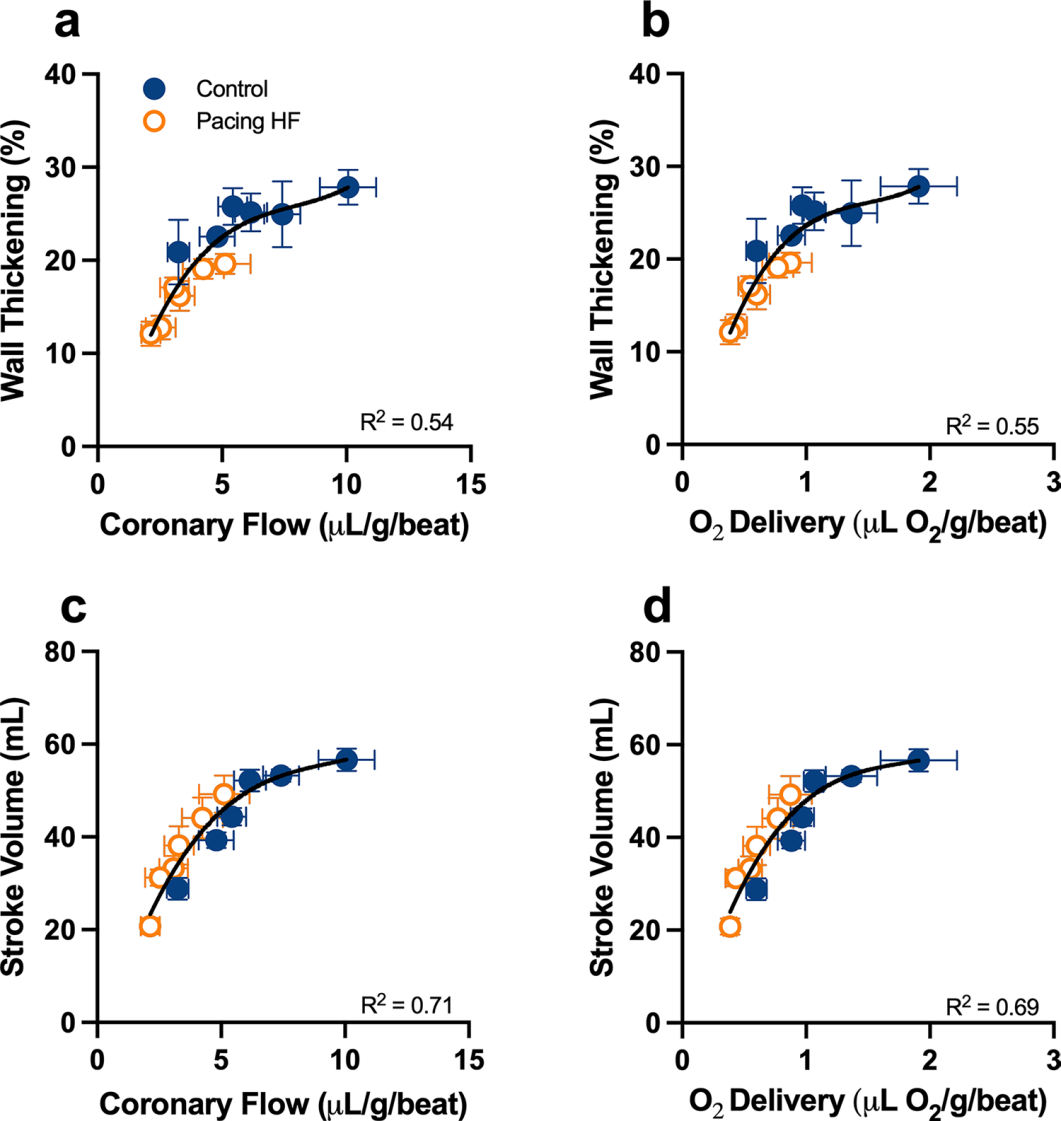
Regional and global indices of cardiac function relative to coronary flow and myocardial oxygen delivery per beat as mean arterial pressure was decreased from ~ 140 mmHg to ~ 40 mmHg in control (n = 5) vs pacing HF (n = 5) swine. **a** Regional systolic wall thickening and **c** stroke volume progressively decreased at coronary flow per beat fell below a threshold volume of ~ 5 µL/g/beat. Similar dependence of **b** regional wall thickening and **d** stroke was also observed as myocardial oxygen delivery per beat fell below an apparent threshold of ~ 1 µL O_2_/g/beat

Figure 4 demonstrates the effect of coronary flow and oxygen delivery normalized per beat on indices of regional and global cardiac function as mean arterial pressure (i.e. CPP) was decreased from ~ 140 mmHg to ~ 40 mmHg. Reductions in regional wall thickening were strongly associated with reductions in coronary flow per beat (Fig. 4a) and the volume of oxygen delivered per beat (Fig. 4b) in both control and pacing HF swine. Similar dependence of LV stroke volume on coronary flow (Fig. 4c) and oxygen delivery (Fig. 4d) per beat was also apparent. Diminished regional and global contractile function of both control and pacing HF swine was highly related to coronary flow and oxygen delivery falling below apparent critical thresholds of ~ 5.0 μL/g/beat and ~ 1.0 μL O_2_/g/beat, respectively, across all levels of systemic pressure.

## Discussion

The purpose of this study was to investigate interrelationships between coronary blood flow, oxygen delivery and consumption, and regional contractile function in heart failure and to investigate potential mechanisms involved. We tested the hypothesis that heart failure attenuates coronary vasodilator and autoregulatory capacity and that deficits in function are directly related to reductions in the volume of myocardial perfusion and oxygen delivered per beat. We used a model of pacing-induced heart failure in Ossabaw swine [12] to demonstrate functional and metabolic consequences of diminished myocardial oxygen delivery per beat in heart failure. The major novel findings of the study include: 1) Swine that were paced possessed phenotypic features consistent with heart failure (Table 1), including elevated LV end diastolic pressure, normal ejection fraction, and reduced coronary blood flow and oxygen delivery at rest (Table 1). 2) Coronary vasodilation to pharmacological and metabolic (pacing) stimuli were significantly reduced by heart failure (Fig. 1). 3) Heart failure impaired coronary pressure-flow autoregulation (Fig. 3), as well as systolic and diastolic function (Fig. 2). 4) Regional and global cardiac contractile function were related to coronary flow per beat and oxygen delivery per beat with apparent thresholds, below which the deleterious effects of heart failure were apparent (Fig. 4). These findings indicate that adaptations in the coronary microcirculation in HF attenuate coronary metabolic and autoregulatory capacity and that subsequent functional deficits are related to reductions in the volume of myocardial perfusion and oxygen delivered per beat.

### Coronary microvascular dysfunction and heart failure

Findings on impaired pharmacologic (Regadenoson) coronary vasodilation in our swine model of pacing HF (Fig. 1b) are consistent with a growing body of evidence of diminished coronary flow reserve in human subjects with heart failure [1, 8, 32, 33]. While diminished flow reserve to adenosine-based analogs is strongly associated with poor clinical outcomes [13, 34–36], distinct differences in baseline and/or “maximal” flow values confound interpretation and potential insight, especially into stand-alone measures of coronary flow reserve [37]. Furthermore, the extent to which this coronary microvascular dysfunction represents a cause and/or consequence of diminished systolic or diastolic function and overt failure remains an ongoing chicken vs. the egg argument across the field [6, 8, 38]. We argue impaired coronary vasodilation in response to a specific metabolic challenge (e.g. increased heart rate; Fig. 1d) likely represents a more patho-physiologically relevant assessment of coronary microvascular responsiveness. Importantly, reductions in pacing-induced increases in coronary blood flow in our model of heart failure are consistent with clinical studies that reported reductions in coronary responses to dobutamine [24] and exercise [39]. Prior data from the Bache laboratory in a dog model of pacing HF indicate decreased coronary flow and metabolic vasodilation in the failing heart reflect impairments in myocardial metabolism and the coupling between cardiac function and MVO_2_ (mechano-metabolic efficiency) [40]. Accordingly, it is important to recognize that reductions in perfusion may represent impairments in myocardial oxygen utilization (metabolism) rather than inadequate delivery of oxygen. Whether primary impairments in perfusion or metabolism contribute to divergent HFpEF-like phenotype in paced Ossabaw swine [12] vs. HFrEF-like phenotype in paced dogs [40–43] remains to be determined.

### Coronary pressure-flow autoregulation and heart failure

To our knowledge, this study is the first to interrogate the impact of heart failure on coronary pressure-flow autoregulation. Pacing HF resulted in marked increases in coronary resistance, decreases in coronary blood flow (Table 2), and diminished autoregulatory capacity over CPPs ranging from 120 to 60 mmHg (Fig. 3). These data support the hypothesis that reductions in pharmacologic coronary flow reserve (microvascular function) extend to functional physiological impairments in coronary flow control in response to critical limitations in perfusion pressure (Fig. 3) [4, 23, 25, 44]. Diminished autoregulatory capability would act to augment the onset of myocardial hypoperfusion, contractile dysfunction, and ischemic injury in the failing heart, especially in the subendocardium where autoregulatory behavior is weakest [45, 46]. Although transmural coronary flow was not assessed in this investigation, reductions in regional systolic wall thickening, which are largely dictated by decreases in subendocardial blood flow [45, 47, 48], support subendocardial perfusion was likely compromised in pacing HF swine (Fig. 3b). While we acknowledge that transmural heterogeneity confounds interpretation of globally measured variables [49], it is intriguing that reductions in coronary flow and contractile function in pacing HF swine occurred in the absence of overt myocardial lactate release and with little/no difference between coronary venous PO_2_ and myocardial oxygen extraction between groups (Table 2). If coronary venous PO_2_ is presumed to reflect myocardial tissue PO_2_ [14, 50], then similar values of coronary venous PO_2_ in control and pacing HF swine (Table 2) indicate that impaired autoregulation in heart failure is most likely related to structural changes, augmented myogenic tone, and/or responsiveness opposed to the attenuation of local metabolic (oxygen-sensitive) control [29, 30]. Additionally, there is a growing body of evidence implicating a functional role for pericytes in coronary autoregulatory behavior that merits future investigation [51].

### Functional consequences of coronary microvascular dysfunction

Our laboratory recently demonstrated that impaired control of coronary blood flow reliably decreases contractile function in proportion to reductions in the volume of myocardial perfusion per beat [16]. Data from this investigation are consistent with this observation for both regional (Fig. 4a) and global (Fig. 4c) indices of cardiac function across CPPs ranging from 140 to 40 mmHg in control and pacing HF swine. Accordingly, lower levels of contractile function in pacing HF were dependent on the degree to which flow fell below an apparent critical threshold of ~ 5.0 μL/g/beat. Ultimately, levels of regional wall thickening (Fig. 4b) and LV stroke volume (Fig. 4d) appear to be dictated by the volume of myocardial oxygen delivered per beat (below threshold of ~ 1.0 μL O_2_/g/beat), as recently proposed by Canty and Weil [26]. These findings indicate that coronary microvascular dysfunction (i.e. oxygen delivery) “causally” contributes to contractile dysfunction in the failing heart. Proportionate reductions of coronary blood flow (Fig. 3a), wall thickening (Fig. 3b) and MVO_2_ (Fig. 3c) in the absence of myocardial lactate release or changes in myocardial oxygen extraction suggest that pacing HF is associated with moderate ischemia that reaches a new, lower set point of myocardial perfusion-contraction-metabolism matching similar to hibernating myocardium [26, 52]. However, reductions in net lactate uptake at lower CPPs in both groups of swine (Table 2) may reflect subendocardial lactate production [53]. Definitive evidence to demonstrate functional and metabolic limitations in the failing heart are “caused” by decreases in flow and oxygen delivery require additional experiments to demonstrate that deficits in wall thickening and MVO_2_ are corrected by the restoration of coronary flow to normal control levels [16, 50, 54]; e.g. cannulated coronary preparation which was not utilized in this investigation. However, similar levels of coronary flow (~ 0.37 mL/min/g vs. ~ 0.33 mL /min/g) observed in pacing HF swine at CPP of 140 mmHg and control swine at CPP of 40 mmHg resulted in comparable values of both wall thickening (~ 20%) and MVO_2_ (~ 34 µL O_2_/min/g) (Table 2; Fig. 4).

### Limitations of the study

Our model of pacing HF in Ossabaw swine provides a unique HFpEF-like phenotype (elevated LV end diastolic pressure with normal ejection fraction) that lies in stark contrast to previous studies in other animal models [40–43] of high-rate pacing, which present more of a dilated HFrEF phenotype. While we submit that our model offers a novel experimental platform on which to interrogate patho-physiologic mechanisms of heart failure, we acknowledge that it does not fully reflect the variability and complexity of the clinical HFpEF syndrome in humans [1, 7]. To this end, it is intriguing that coronary flow (myocardial oxygen delivery) was increased at rest in our recent study of pacing HF in lean Ossabaw swine [12] versus significantly reduced in the present study (Table 1). Reasons for this discrepancy are unclear but could be related to underlying differences in genetic predisposition of Ossabaw swine included in each study [55]. Mechanisms of chronotropic incompetence were not assessed in this study but have been attributed to a variety of pathways, including desensitization or downregulation of beta-adrenergic signaling, impaired baroreflex response, and SA nodal dysfunction [1, 56–59]. Due to limitations of the open-chest echocardiographic approach, we were unable to consistently acquire reliable measures of regional (stress–strain) or global (E/A and E/e’) indices of diastolic function. Thus, future studies are required to specifically address the extent to which reductions in myocardial perfusion and oxygen delivery per beat determine the degree of diastolic dysfunction in failing hearts. Further studies to assess the state of myocardial energetics (high-energy phosphates) in the absence and presence of controlled coronary flow conditions are also warranted to examine whether this steady hemodynamic state of pacing HF simply reflects overt ischemic contractile dysfunction or a more adaptive state of chronic myocardial hibernation [60–62].

We acknowledge that sodium nitroprusside is a potent coronary vasodilator that would act to increase coronary blood flow, diminish overall autoregulatory capacity, and contribute to hibernation by reducing MVO_2_ and preserving contractile function [63–65]. However, the concentrations that were used in the present experiments to reduce CPP were relatively low and do not appear to have appreciably diminished autoregulation as autoregulatory gain in control swine averaged 0.86 ± 0.24 (consistent with strong autoregulatory response; Fig. 3a). The use of phenylephrine to increase CPP is unlikely to influence autoregulation as alpha-adrenergic activation has little effect on the coronary circulation of swine [66].We recognize that declines in regional contractile performance are primarily driven by reductions in subendocardial blood flow [45, 47, 48], which we did not measure in the present study. This limitation is partly offset by the minimal collateral blood flow and by the fact that, in swine, transmural measurements of regional contractile function and perfusion are weighted toward the subendocardium [67]. In addition, transmural flow heterogeneity complicates the interpretation of global measures such as MVO₂, myocardial oxygen extraction, and lactate uptake or release, because these parameters differ between the subepicardial and subendocardial layers [49]. While prior studies suggest that elevated LV pressures contribute to the promotion of subendocardial ischemia [68–70], the extent to which increased LV end diastolic pressure is responsible for the phenotype of pacing HF swine remains to be determined. We submit that the low levels of myocardial oxygen extraction are most likely due to the anesthetized open-chest preparation utilized for this investigation, as previously observed by our laboratory [16, 30]. Further clarification of the apparent regional and condition-dependent variability in the complex interplay between myocardial perfusion, contractile function, and metabolism, particularly in heart failure, will require additional experimental manipulation, measurements, and larger datasets.

### Implications and conclusions

Taken together, our findings support that adaptations in the coronary microcirculation in the setting of pacing HF attenuate coronary metabolic and autoregulatory capacity and that subsequent functional deficits are directly related to reductions in the volume of myocardial perfusion and oxygen delivered per beat. These results have several key implications for the interpretation of coronary microvascular dysfunction in heart failure and for the design of future studies and therapies. Our findings define relative threshold values of coronary flow and oxygen delivery per beat below which functional and metabolic deficits become apparent. Second, the observed perfusion–contraction–metabolism matching supports a causal chain in which coronary control abnormalities (enhanced myogenic tone and vessel stiffening) directly shape myocardial perfusion and the subsequent degree of contractile dysfunction. Finally, our results highlight the need for clinical studies to quantify coronary flow and oxygen delivery normalized to cardiac cycle (µL/g/beat) in relation to measures of regional myocardial function and energetics throughout the initiation and progression of the heart failure phenotype. We postulate that viewing coronary control in terms of per‑beat delivery, rather than pressure or flow reserve alone, could prove essential for identifying specific conditions and individual subjects where the coronary microcirculation represents a causal target for therapies to restore effective beat‑to‑beat perfusion.

## Data Availability

The data that support the findings of this study are openly available via figshare at: 10.6084/m9.figshare.31839244. Further enquiries can be directed to the corresponding author.
